# Physician Burnout Through the Female Lens: A Silent Crisis

**DOI:** 10.3389/fpubh.2022.880061

**Published:** 2022-05-24

**Authors:** Hemasree Yeluru, Heather L. Newton, Rupa Kapoor

**Affiliations:** ^1^Eastern Virginia Medical School, Norfolk, VA, United States; ^2^Children's Hospital of the King's Daughters, Norfolk, VA, United States

**Keywords:** burnout, resilience, COVID, female physician, risk factors, solutions

## Abstract

Physician burnout, the emotional exhaustion and depersonalization that arises from job fatigue and dissatisfaction, is a rapidly growing problem. Although burnout has been a recognized problem for decades, our healthcare system has yet to devise a sustainable solution. Additionally, burnout does not affect all physicians in the same way- women physicians have disproportionately higher rates of burnout than male physicians. Burnout poses a tremendous risk to our public's health with its severe and debilitating effects on both physician and patient health alike. We must intervene as early as medical school and residency at both the systemic and individual levels to combat burnout. Clinical leadership training might be one sustainable approach to begin addressing burnout in female physicians.

## Introduction

Each year upwards of fifty thousand brilliant college students apply to medical school^1^ to begin their laborious journey to yield the power of healing. Driven by an intrinsic calling to serve, these students assent to nearly a decade of immensely demanding post-graduate training. Students often survive these grueling years by focusing on the light at the end of the tunnel: becoming an attending physician. The long hours, sleepless nights, high workload, and emotionally taxing patient scenarios are all justified by the promise of being an attending; to finally be the leader, the teacher, the healer. However, many of these unsuspecting physicians are struck with reality as they transition to the clinical environment.

Physicians today are exhibiting alarming rates of burnout and job-related fatigue. Burnout, defined by the Maslach Burnout Inventory (1), is a psychological syndrome of emotional exhaustion, depersonalization, and reduced personal accomplishment that can occur among individuals who work with other people in some capacity. Physicians, when compared to those in other highly demanding professions, have over twice the rate of burnout and work stress (2). How did this problem arise? A public health emergency can be defined as a situation where health consequences have the potential to overwhelm a community's routine capabilities to address them (3). This very pattern is unfolding in our current healthcare system. A 2019 review (4) found that 44% of physicians report symptoms of burnout, and an unsettling 14% reported suicidal ideations. With more than a third of physicians feeling overworked, fatigued, and unsatisfied, there is a risk to the health and safety of our patients.

There are several unique internal and external pressures physicians' face that contribute to burnout in medicine. Sleep deprivation, unhealthy coping mechanisms, lack of self-care, lack of social support, and self-critical tendencies are more prevalent amongst physicians (5). Systemic factors such as high patient loads, poor compensation, lack of benefits or paid-time-off, and limited opportunities for personal advancements also contribute to burnout (6). The growth of electronic medical record (EMR) systems increased the administrative burden on physicians. In 2018, it was found that for every hour spent with a patient, a physician spent two additional hours of uncompensated time performing administrative duties such as writing notes, ordering medications, and documenting procedures (7). Many of these administrative tasks are completed at home, further limiting the time a physician has for self-care and family. The above causes of physician burnout are well-described in the literature. Additionally, there has been a call for systems level interventions to address burnout, such as decreasing administrative burden, providing resources for wellness and resilience, aligning organizational values and allowing for flexible schedules (8). There is a dearth of literature on what measurably impacts burnout, resulting in a further call for research on impact of interventions (9). Interventions that appear to make a difference include improving teamwork, flexible or creative schedules, and improving workflow and technology ^2^.

Burnout cannot be approached in a standard fashion for all physicians; its manifestations vary vastly across physicians of different genders, ages, specialties, and race. Exceptionally striking is the statistic that burnout in female physicians may be up to 60% greater than that of male physicians (10), with women scoring higher for emotional exhaustion and men scoring higher for depersonalization (11). Influencing factors may include the unique challenges many women physicians face including responsibility for family needs, role in child-bearing, infertility, inadequate compensation, lack of leadership opportunities, scarce mentorship, and decreased recognition and pay when compared to their male counterparts (12). Despite these challenges, one study notes that female physicians have significantly better patient outcomes than do male physicians (13). Based on AAMC and AMA database and survey data in 2020, 36% of the physician workforce identified as female; this percentage is expected to reach 50% over the next few decades (14). Thus, as the ratio between male and female physicians equalizes, we must uplift women physicians by mitigating the unique pressures they face, not just for the physician's sake, but also for the patients'. The first step is to understand the specific consequences resulting from burnout on the health of physicians, patients, and our community as a whole.

## Why Is Burnout A Problem?

“Illness doesn't belong to us. It belongs to them. Doctors need to be taught to be ill. We need permission to be ill and to acknowledge that we are not superhuman” (15). Spoken over two decades ago, this sentiment still holds true amongst many physicians. Driven by unrelenting dedication to their patients, it is easy for physicians to turn a blind eye to their own challenges. However, burnout is a force to be reckoned with; an illness with devastating long-term consequences for the health of our physicians, patients, and community. There is a strong correlation between burnout and the deteriorating mental and physical health of physicians today. In 2019, the National Institute of Mental Health (16) found that 8.5% of all US adults experienced at least one episode of major depression while a startling 20% of practicing US physicians struggle with depression (17). There is little data on how many physicians seek out treatment for their depression, and regardless, many sources theorize that this statistic would be underreported. If it is true that very few physicians receive help for their depression, it may explain their alarming rates of suicidal ideation (18). Of note, male physician suicide rates are lower than that of non-physician males while female physician suicide rates are higher than that of non-physician females. For context, rates of suicide in non-physicians are greater for males than females (19), making the higher rate of female physician suicide even more disturbing.

Physicians also suffer from chronic medical illnesses such as hypertension, high cholesterol, diabetes, obesity, autoimmune disorders, and chronic pain (20). Many of these personal medical problems are swept under the rug for the sake of their career. Women physicians experience unique health concerns as a result of burnout, several stemming from the inopportune co-incidence of childbearing years and their grueling early-career. The average age of matriculating medical students in 2018 was 24, meaning 50% of physicians complete their medical training after their mid- 30s depending on their chosen specialty (21). Advanced maternal age (22) is considered >35 years and is associated with higher rates of infertility and pregnancy complications (23). In a 2019 survey, 25% of women physicians reported issues with fertility and 79% of them pursued treatment (24). Amongst women surgeons the data is even more striking; 42% of female surgeons reported struggling with fertility and pregnancy loss (25). In comparison, the infertility rate in non-physician women is 10% (26). Seventy two percent of women associate the demands of their medical training and work-related fatigue to their obstetric complications (24). Fertility is just the first step. What happens after becoming pregnant? Between diagnosis of infertility, undergoing treatment, and successfully becoming pregnant we have added additional years to the age of physicians. Women of advanced maternal age experience significantly more pregnancy complications: restricted fetal growth, preeclampsia, placental abruption, preterm labor, miscarriages, and stillbirth–all outcomes that affect a mother's physical and mental health (23). This creates an onerous fork in the road for women physicians: is it better to delay pregnancy until after completing training or to have a child as early as possible to ensure good health? There is a lack of institutional and policy support in medicine (long work hours, night shift, prolonged standing) that is known to have detrimental health effects on pregnant women. Then comes the next question, what happens after starting a family? Women physicians carry the majority of household duties related to groceries, cooking, cleaning, children's schooling, activities, and doctor's appointments compared to their male colleagues (27). Juggling childbearing and managing a household along with long work hours, sleepless nights, high patient loads, and emotionally charged patient scenarios is a setup for burnout unless proper institutional policies along with individual resilience techniques are put in place.

Despite physicians' steadfast commitment to “do no harm,” burnout is a major concern when it comes to patient safety. A 2014 survey found that 10.5% of physicians had made at least one major medical error in the past 3 months, 77% of whom also reported symptoms of burnout and decreased quality of life (28). However, sound medical care sits on a spectrum of adequacy and is not so simple to allow this conversation to rest at error vs. not. Medicine is nuanced and requires a significant amount of personal judgment on the physicians' behalf. The depersonalization and emotional exhaustion associated with burnout can cloud the decision making ability of physicians. We must work to prevent and reverse symptoms of burnout in physicians to protect the safety of our patients.

Lastly, physician burnout also has unacceptable consequences on our community as a whole. Our healthcare system is facing a mass exodus of physicians; an estimated 40,000–90,000 physicians are estimated to leave the workforce by 2025 (29). Physician shortage means higher turnover, and higher turnover means higher costs. Healthcare in America is already treading murky waters, and this inevitable physician shortage will only further strain our healthcare system and ultimately harm patients and our communities. To make matters worse, the shortage is more striking in front-line specialties such as primary care (internal medicine, pediatrics, family medicine), emergency medicine, and general surgery- fields that are crucial to manage our chronically ill and most expensive patients. David Mirvis, a healthcare economist, notes that more medical students are applying to competitive specialties rather than primary care, which will worsen the physician shortage (30). He describes this as an “incongruence between what medical students want and what society needs.”

Of note, burnout amongst primary care physicians is disproportionately high when compared to other specialties. In addition to the workload due to physician shortages in these specialties, one theory is that frontline physicians are more acutely aware of the socio-economic determinants of poor health, and this creates a sense of “powerlessness” that leads to a distinct and uncommon pillar of burnout: futility (31). Primary care specialties attract more women physicians and this could be another factor for the higher rates of burnout in female physicians. This is yet another reason to focus on preventative techniques specific to women physicians.

## Effects of COVID-19

The COVID-19 pandemic couldn't have attacked at a more inopportune time; if only our healthcare system could evolve as quickly as a virus. The pandemic has devastated the emotional capacity and resilience of our frontline healthcare workers. Rates of anxiety, depression, burnout, job fatigue, and emotional drain are higher than ever (32). It is too soon to know the extent to which this has accelerated the number of physicians leaving medicine, but it is expected that the burnout caused by COVID-19 could be a tipping point in that decision. Work-related stressors included pay-cuts, working overtime, isolation from peers, and even losing their jobs. At home, physicians were isolated from their families for fear of spreading the disease which limited a major burnout prevention strategy—connecting with others—in a time when it was most needed. Additionally, the pandemic further widened the gap for women physicians' secondary to an increase in household responsibilities and shortage of childcare (33). Our healthcare system was in no shape to handle a pandemic of this gravity at baseline and inevitably suffered due to the lack of institutional and individual support for physicians, especially female physicians.

One positive outcome of the pandemic, however, was the widespread use of tele-medicine across specialties. New York City's NYU Langone Health, where COVID-19 was especially rampant, found that there was an 8-fold increase in the usage of tele-medicine visits after the start of the pandemic (34), which allowed non-critical patients to have regular care and follow-up with their doctors in a safe manner. The prompt implementation of tele-medicine was essential for physicians to counter the demanding nature of the pandemic and still ensure the safety of their patients. The use of tele-medicine also reduced patient flow to hospitals which alleviated some work burden and allowed front line physicians to focus on their critically-ill patients thereby avoiding overwork, fatigue and burnout (35).

## Current Solutions

Burnout is a deadly disease with consequences pervading every part of our healthcare system. Unless we implement equally potent solutions, we will be no match for this pervasive epidemic. As the most effective solutions are always multifaceted, the approach must combine both systemic and individualized strategies. In 2017, the Accreditation Council of Graduate Medical Education (ACGME,) which governs the standards for medical education (36), acknowledged the growing problem of physician burnout by adding a “Well-being” requirement for all residencies. This obligated programs to include wellness initiatives and burnout mitigation measures as a contingency for accreditation. This top-down approach to burnout is important because it sets minimum standards early in training. The onus on individual programs to create, implement, and evaluate interventions-will require time and due diligence if they are to do more than “check a box.”

Ideally, institutions would put in place systemic changes to address underlying causes of burnout. In a system strained by workforce shortages, finances, and regulatory burdens, addressing burnout has not been a priority and will require time. In the meantime and in addition, we have to find a way to build and support individual physicians without blaming them for this systemic problem. Some institutions have explored the concept of promoting resilience, defined as the ability to “bounce back” after an adverse event and respond to stress in a way that causes minimal psychological and physical cost to oneself (37). Resilience is a crucial skill set for physician well-being and patient safety. One theory is that clinical leadership, the ability to effectively lead and coordinate teams, communicate clearly, model positive behaviors, and show emotional intelligence, could be the tipping point between burning out and remaining resilient (38). The skillset for leadership and resilience have significant overlap (39). Physicians who lack leadership training are more likely to burnout and have negative impacts on their ability to provide patient care (8).

Other, more individualized solutions that have been implemented into the workplace include: stress management workshops, improved diet and exercise regimens, mental health treatment, spiritual counseling, mentorship, leadership opportunities, work-life integration, help with child-care, and flexible schedules. One could postulate the specific benefits of each of these interventions specifically for women physicians given the data above on disproportionate burden of household duties and the lack of leadership opportunities (Table 1). Specifically for women, there are some interventions include improved maternity leave policies and support during lactation. Extra benefits during the perinatal period can have tremendous positive effects on maternal mental health and experiences with burnout (40).

**Table 1:** Specific risk factors contributing to female physician burnout and potential remedies.

	**Specific Risk Factors**	**Remedies**
Workplace	Unequal pay Lack of mentorship Limited leadership opportunities Time constraints	Closing the pay gap Structured mentoring programs focusing on challenges of female physicians Clinical leadership training in early career Transparent advancement policies Diversity and inclusion measures for gender inequity in leadership Flexible scheduling Salaried time off Consideration of timing and scheduling of key stakeholder meetings
Home	Increased household responsibilities relative to male counterparts Increased childcare responsibilities relative to male counterparts	Meal preparation services Laundry services Onsite daycare Hazard hours child care assistance Sick care assistance Assistance with child care transportation needs
Health	Childbearing Increased maternal age Mental health	Improved maternity leave policies Flexible schedules and considerations for unique workplace stressors (prolonged standing/hours, night shift differential) Timing/scheduling for prenatal visits Easy access to Mental Health Services, hazard hours availability Subsidized access for trainees

## A Call To Action

The dedication necessary for medicine is an intrinsic quality that lies deep within physicians, it is not a trait that can be taught. As cheesily written in countless medical school applications, it truly is one's “calling.” Relying on the inseparable bond of trust between patients and physicians, medicine requires a high degree of emotional intelligence in order to be successful. Our current medical system has weaponized burnout and alienated physicians from their life's passion. We must work together to address burnout in our physician workforce; we owe it to our patients and community. Our current model of medical education is based on the Flexner Report written in 1910 (41). It created a separation between medicine and public health that, despite 112 years passing since its inception, still persists. However, with today's highly interdependent society, medicine and public health cannot be considered distinct entities, but only two sides of the same coin. Physician burnout has proven to be the epitome of this paradigm with its deleterious effects not only on physician health but that of patients and communities.

To protect future generations of physicians, especially women, we must incorporate holistic, intentional interventions into the workplace, and it will not be an easy fight. Women must organize and challenge current norms including but not limited to: unequal pay, lack of recognition, limited opportunity for scholarship, decreased promotions, and few leadership roles. We call on our sisters in healthcare to work together to reduce burnout and uplift our physician workforce. We need to incorporate clinical leadership and resilience training for all physicians- not just the ones at the male-dominated senior career level. In fact, it is never too early to start leadership and resilience training. At our institution, we took burnout into our own hands and began working with residents. We hold monthly lunch hour sessions where residents come together and enjoy an uninterrupted hour of conversation and skill building around challenges in the workplace, managing job stress, work-life integration, and overcoming symptoms of burnout. These sessions are extremely popular amongst residents and insightful for all.

By not just acknowledging but also addressing burnout early in a physician's career, we hope to be one step ahead of this epidemic. Young physicians deserve medical education that values their being and promotes wellness to set them up for success throughout their career. Residency does not have to remain antiquated like Flexner had intended- with overworked and unhappy physicians. Medicine is filled with miracles and has utmost potential to bring joy to both the provider and patient; let us not lose that to burnout. Recently in a refreshing conversation with a physician she was asked, “How do you achieve work-life balance?” She replied without a moment's hesitation, “I don't strive for work-life balance because I love my work.”

## Data Availability Statement

The original contributions presented in the study are included in the article/supplementary material, further inquiries can be directed to the corresponding authors.

## Author Contributions

HY, HN, and RK contributed to the conception and design of this paper and all participated in writing sections of the manuscript. HN and RK helped to draft the outline and HY took the lead on the first draft of the manuscript. All authors contributed to the manuscript revision, read, and approved the submitted final version.

## Conflict of Interest

The authors declare that the research was conducted in the absence of any commercial or financial relationships that could be construed as a potential conflict of interest.

## Publisher's Note

All claims expressed in this article are solely those of the authors and do not necessarily represent those of their affiliated organizations, or those of the publisher, the editors and the reviewers. Any product that may be evaluated in this article, or claim that may be made by its manufacturer, is not guaranteed or endorsed by the publisher.

## Footnotes

^1^Available online at: https://www.aamc.org/news-insights/medical-school-applicants-and-enrollments-hit-record-highs-underrepresented-minorities-lead-surge#:~:text=Total%20applicants%20for%202021%2D22/2002%2D03%3A%2085.7%25

^2^Available online at: https://www.ama-assn.org/practice-management/physician-health/physician-burnout-4-types-interventions-and-how-they-can-help
